# A Multiwall Path-Loss Prediction Model Using 433 MHz LoRa-WAN Frequency to Characterize Foliage’s Influence in a Malaysian Palm Oil Plantation Environment

**DOI:** 10.3390/s22145397

**Published:** 2022-07-20

**Authors:** Rabeya Anzum, Mohamed Hadi Habaebi, Md Rafiqul Islam, Galang P. N. Hakim, Mayeen Uddin Khandaker, Hamid Osman, Sultan Alamri, Elrashed AbdElrahim

**Affiliations:** 1 IoT & Wireless Communication Protocols Lab, Department of Electrical and Computer Engineering, Kulliyyah of Engineering (KOE), International Islamic University Malaysia (IIUM), Kuala Lumpur 53100, Malaysia; rabeyaanzum@gmail.com (R.A.); rafiq@iium.edu.my (M.R.I.); galang.persada@live.iium.edu.my (G.P.N.H.); 2 Department of Electrical Engineering, Faculty of Engineering, Universitas Mercu Buana, Jakarta 11650, Indonesia; 3 Centre for Applied Physics and Radiation Technologies, School of Engineering and Technology, Sunway University, Bandar Sunway 47500, Malaysia; mayeenk@sunway.edu.my; 4 Department of General Educational Development, Faculty of Science and Information Technology, Daffodil International University, DIU Rd, Dhaka 1341, Bangladesh; 5 Department of Radiological Sciences, College of Applied Medical Sciences, Taif University, P.O. Box 2425, Taif 21944, Saudi Arabia; ha.osman@tu.edu.sa (H.O.); s.alamri@tu.edu.sa (S.A.); rashidrahim1976@yahoo.com (E.A.)

**Keywords:** multiwall model, path loss, foliage loss, LoRaWAN, 433 MHz, smart agriculture

## Abstract

Palm oil is the main cash crop of tropical Asia, and the implementation of LPWAN (low-power wide-area network) technologies for smart agriculture applications in palm oil plantations will benefit the palm oil industry in terms of making more revenue. This research attempts to characterize the LoRa 433 MHz frequency channels for the available spreading factors (SF7-SF12) and bandwidths (125 kHz, 250 kHz, and 500 kHz) for wireless sensor networks. The LoRa channel modeling in terms of path-loss calculation uses empirical measurements of RSS (received signal strength) in a palm oil plantation located in Selangor, Malaysia. In this research, about 1500 LoS (line-of-sight) and 300 NLoS (non-line-of-sight) propagation measurement data are collected for path-loss prediction modeling. Using the empirical data, a prediction model is constructed. The path-loss exponent for LoS propagation of the proposed prediction model is found to be 2.34 and 2.9 for 125–250 kHz bandwidth and 500 kHz bandwidth, respectively. Again, for the NLoS propagation links, the attenuation per trunk is found to be 7.58 dB, 7.04 dB, 5.35 dB, 5.02 dB, 5.01 dB, and 5 dB for SF7-SF12, and the attenuation per canopy is found to be 9.32 dB, 7.96 dB, 6.2 dB, 5.89 dB, 5.79 dB, and 5.45 dB for SF7-SF12. Moreover, the prediction model is found to be the better choice (mean RMSE 2.74 dB) in comparison to the empirical foliage loss models (Weissberger’s and ITU-R) to predict the path loss in palm oil plantations.

## 1. Introduction

Long-range low-power wide-area network (LoRa LPWAN) technology is unique and remarkable technology because of its long-range coverage, low power consumption, and low-cost system architecture. These features have allowed LoRa LPWAN to become a favorable option for performing communications in most IoT (Internet of things) wireless applications for smart agriculture [1]. The vision of smart farming comes with the application of modern IoT technologies for autonomous monitoring, data processing, and decision making [2]. New, challenging situations are arising for the deployment of LoRa in farmlands, due to the diverse characteristics of the agriculture farms and foliage loss caused by the plants [3]. Palm oil is the main cash crop of Malaysia, and most of the farmland in Malaysia is devoted to palm oil plantations. The use of LoRa-IoT applications in palm oil plantations can bring a revolutionary improvement in the palm oil industry through smart farming activities; however, LoRa data transmission is vulnerable due to the foliage blockage [4] caused by vegetation canopies [5]. Palm oil plantations are covered by oil palm trees, which are planted in rows. Moreover, tree foliage is very prone to absorbing water, causing scattering of the propagated signal. Low frequencies, such as 240 MHz (compared to 700 MHz), are less prone to being affected by weather conditions such as rain and strong wind [6]. However, the tree foliage causes obstruction of the propagation path, and foliage obstruction loss is unavoidable in an agricultural farm. Furthermore, the plantation area with matured oil palm trees is densely foliated and can cause a significant propagation loss due to its pattern. Although data transmission loss due to obstruction in the propagation path can be solved using a routing scheme [7,8,9], significant propagation loss remains due to the dense foliage. Taking the advantage of the symmetric pattern of the oil palm trees in the plantation area to solve this problem, in this study we propose a new path-loss model. Therefore, this research attempts to analyze the LoRa LPWAN propagation channels using 433 MHz frequency in a palm oil plantation located in Selangor, Malaysia, using empirical measurements. Hence in this research, a prediction model is initiated and compared to the existing empirical models. The contributions of this article are as follows:

- A multiwall path-loss model for palm oil plantation foliage is proposed.
- Path-loss prediction is modeled for LoRa LPWAN 433 MHz frequency channels.
- The proposed and predicted path-loss models are compared with existing empirical models.

## 2. Related Work

In recent years, a lot of research has been carried out on path-loss estimation for roadside trees, single trees, lines of trees, etc., based on empirical models. An empirical measurement and path-loss modeling using RFID (radio frequency identification) propagation at 433 MHz was carried out in a palm oil plantation in Malaysia [10]. The average loss for a single oil palm tree using an RFID signal was found to be 2.49 dB, and Weissberger’s exponential decay model showed almost the same results for the measured propagation path loss. To characterize the LoRa propagation path loss in a tropical vegetative environment, empirical research was conducted, concluding that the tree canopy area caused the greatest loss of about 56 dB [11]. The measurements were performed in the vegetation area of mixed forest, utilizing a LoRa 868 MHz frequency.

Another study was conducted at the 700–800 MHz band through a line of trees in a suburban park in Brazil, focusing on vegetal morphology with varied frequency and antenna height [12]. In this study, the empirical path-loss model was compared to the existing empirical models, showing similarities with Weissberger’s model. Similar approaches were used to predict path loss in different foliage environments.

Utilizing the early ITU foliage path-loss model, a study was conducted to find the error function for foliage depth and develop a tuned model [13]. The results showed that the foliage depth-tuning method had the best prediction performance, with an RMSE of 2.92 dB for the training dataset and an RMSE of 3.71 dB for the validation dataset. Empirical vegetation models were analyzed in vegetation environments including a banana plantation [14], a tomato greenhouse [15], and a coniferous forest [16], showing that the conventional empirical models are not suitable to predict the path loss in vegetated environments, because of the diversity of canopy density. Palm oil plantations comprise a dense canopy, and there is no existing site-specific path-loss prediction model for palm oil plantations. Therefore, there is a need for a site-specific empirical path-loss model for palm oil plantations that can predict the path loss for LoRa LPWAN 433 MHz frequency channels.

## 3. Materials and Methods

In this section, the related empirical foliage models are briefly discussed, after which the proposed model for palm oil plantations is introduced. The other parts contain the measurement environment, oil palm tree specifications, measurement equipment description, and data gathering methodology.

### 3.1. Related Empirical Models

The free-space models are not capable of predicting the channel attenuation due to the presence of foliage obstruction (e.g., tree trunks, leaves, branches, and canopies) in the propagation path [17]. To build an optimal monitoring system, the propagation quality should be examined, and path-loss estimation can be used to predict the channel quality for a certain distance in the propagation field [18].

To predict the loss of propagating signal in foliage, an empirical foliage propagation exponential decay model was initiated. Later, several modified exponential decay (MED) models for low frequencies—namely, the Weissberger model [19] and the ITU recommended model (*ITU-R*) were used [20]. These models were constructed considering the exponential functions, including operating frequencies and path lengths. However, each of these models was subjected to a specific dataset and environmental conditions. Weissberger’s model is applicable for short-distance propagation through the vegetation in the frequency range from 230 MHz to 95 GHz. The expression of the model for *f* frequency and *d* foliage depth is as follows:

(1) $$L_{W}\left(\mathrm{dB}\right)=\{\begin{array}{c} 1.33f^{0.284}d^{0.588}\;;14\;m<d<400\;m\\ 0.45\;f^{0.284}d\;\;;0\;m<d<14\;m \end{array}$$

Other empirical vegetation models for frequencies from 30 MHz to 100 GHz are also capable of prediction signal attenuation at certain vegetation depths. The *ITU-R* model for *f* frequency and *d* foliage depth is expressed as follows:

(2) $$L_{\mathrm{ITU}-R}\left(\mathrm{dB}\right)=0.2f^{0.3}d^{0.6}\;;d<400\;m$$

The *ITU-R* model is optimized by least square error for high frequency ranges (11.2 GHZ to 20 GHZ), where in-leaf and out-of-leaf vegetation is named as fitted FITU-R [19,20]. Another modified version of the *ITU-R* vegetation model is COST-235, designed for the frequency range 2.5–2.7 GHz [21,22]. Since this research concerns 433 MHz frequencies, the Weissberger and *ITU-R* vegetation models are compared with the proposed and predicted models.

### 3.2. The Multiwall Path-Loss Model for Palm Oil Plantations

The multiwall path-loss model has usually been used to analyze indoor electromagnetic wave propagation [23,24,25] from industrial plants [26], laboratory buildings [27], school buildings [28], and many others. This model is derived from one of the basic path-loss models for indoor environments—the one-slope model [29]—where the free-space path-loss term is introduced as follows:

(3) $$PL_{one\;slop}=PL_{0}+10nlog\left(d\right)\;$$

where $PL_{0}=$ reference pathloss, which is the path loss over 1 m distance; *d* is the distance between the transmitter and the receiver; and *n* is the path-loss exponent that indicates how fast the path loss increases with distance.

The path-loss exponent (*n*) defines the rate of power decay with respect to distance [30]. This model can also be developed to become a multi–frequency, multiwall and -floor path-loss model [31]; however, in this research we only used one frequency; therefore, a multi-frequency model was not used in this research.

The RSSI measurements indicate the strength of the radio signals when they reach a specific distance (*d*). Higher RSSI values indicate good signal quality, while low RSSI values indicate that the signal is lost and fails to communicate. Hence, the path-loss exponent is:

(4) $$n=(P_{rd}-P_{r0})/10log_{10}\left(d\right)\;$$

where *n* is the path-loss exponent, *d* is the distance between the transmitter and receiver, $P_{rd}$ is the RSSI at *d* distance, and $P_{r0}$ is the RSSI at 1 m distance [32]. To quantify the intervening wall loss in the multiwall path-loss model, the COST231 multiwall model was proposed by the European Commission in 1999. Therefore, for the same floor environment, the equation of multiwall path loss [23] is:

(5) $$PL_{cost231}=PL_{0}+10nlog\left(d\right)+\overset{M}{\underset{i=1}{\sum }}PL_{i}$$

where *M* is the total number of walls to be traversed in the path along which the radio signal propagates, and $PL_{i}$ denotes the total loss due to the intervening walls [33]. In the palm oil plantation environment, the signal propagation path crosses either trunks or canopies; therefore, the total intervening wall loss can be expressed as follows:

(6) $$\overset{N}{\underset{i=1}{\sum }}PL_{i}=\overset{N}{\underset{C=1}{\sum }}C_{N}+\overset{N}{\underset{T=1}{\sum }}T_{N}$$

where,

$C_{N}\;$= Attenuation caused by *N* number of canopies.
$T_{N}\;$= Attenuation caused by *N* number of trunks.

Therefore, the proposed path-loss model for palm oil plantations is as follows:

(7) $$PL_{palm-oil}=PL_{0}+10nlog\left(d\right)+\overset{N}{\underset{C=1}{\sum }}C_{N}+\overset{N}{\underset{T=1}{\sum }}T_{N}$$

In the path-loss equation, the total canopy and trunk loss can be predicted using the average attenuation caused by a single canopy and trunk, multiplied by the number of trunks and canopies present in between the propagation links.

**Figure 1:**
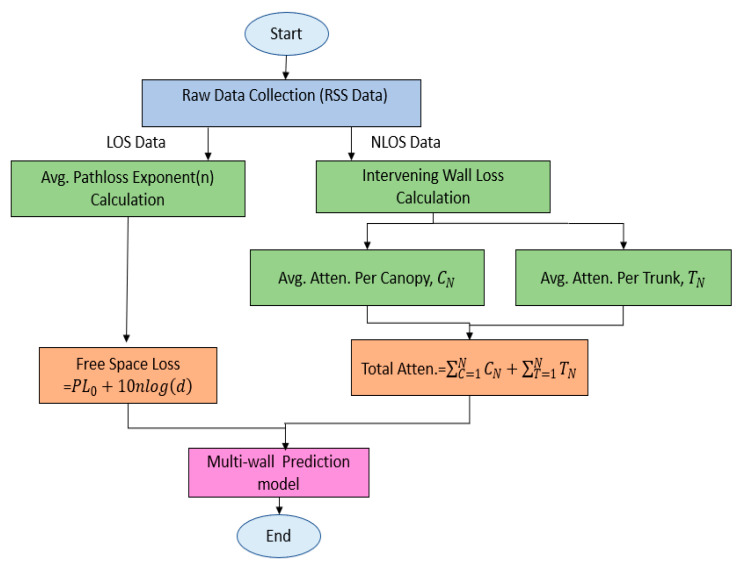
Multi-wall prediction model.

Since this research concerns the LoRa 433 MHz frequency, the proposed path-loss model was utilized for all LoRa channels (SF7-SF12) and three bandwidths (BW 125 kHz, BW 250 kHz, BW 500 kHz) by empirical measurement methods in the palm oil plantation. The following flowchart (Figure 1) concludes the multi-wall path-loss prediction modeling of the palm oil plantation.

### 3.3. Measurement Environment

**Figure 2:**
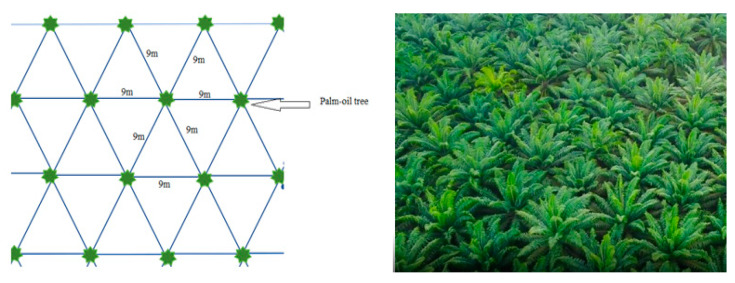
Palm oil planation pattern (**left**); palm oil plantation top view (**right**).

Malaysia, as a typically tropical location, is very popular for its palm oil plantations. To ensure a good harvest, oil palm trees must be planted at certain density. Thus, oil palm tree plantation follows a specific pattern. According to the FAO (Food and Agriculture Organization), the oil palm pegs are planted in straight, lines leaving 7.8 m between rows and 9 m between pegs (Figure 2). Thus, 143 oil palms can be planted per hectare.

Moreover, the adult trees form multiple lines of trees, where the trees can be considered as intervening walls for an NLoS (non-line-of-sight) propagation path in a line of trees. Thus, the pattern of the planted trees in the plantation area can be considered as a multiwall environment where the propagation links cross multiple walls (oil palm trees). Therefore, the palm oil path-loss model can be proposed in line with the multiwall path-loss models.

The measurement site is located at Kuala Kubu Bharu, Selangor, Malaysia. The farmland designated for the experiment is 2 km in length and 0.5 km in width. Measurements were conducted on 15–30 July 2021, and no rainfall occurred during the measurement campaign. From the data of climate briefings (en.climate-data.org), it is noticeable that the average temperature was 26.6 °C (maximum temperature 30.2 °C), and mean humidity was 80%. Moreover, the measurement campaign was put on hold during strong wind conditions. The month of July is part of the dry season in Malaysia, and there was no rainfall for 15 days during the measurement campaign. Therefore, there was no presence of stagnant water on the plantation surface, and the trees’ leaves were not wet.

### 3.4. Oil Palm Tree Specifications

**Figure 3:**
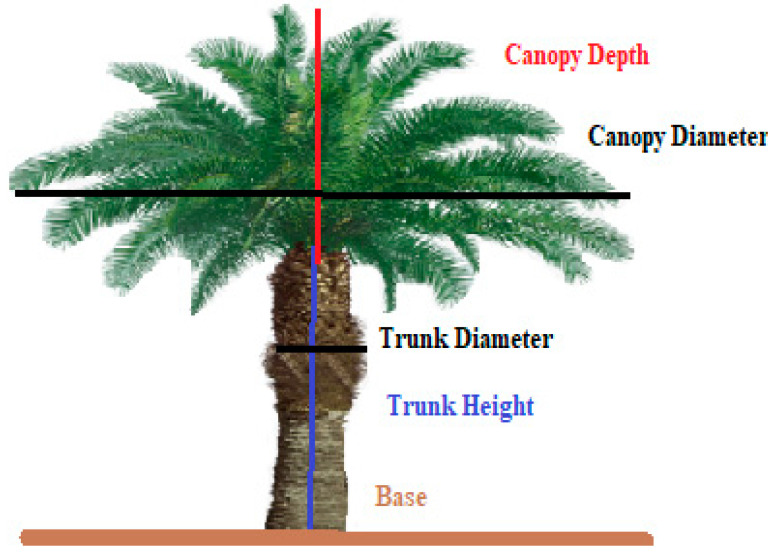
Nomenclature of the oil palm tree.

Palm oil is a very popular source of edible vegetable oil, which is derived from the reddish pulp (mesocarp) of the palm fruit. A specific variety of oil palm tree is known as *Elaeis*, from which palm oil is extracted. The species of oil palm trees include the African oil palm (*Elaeis guineensis*), American oil palm (*Elaeis oleifera*), and maripa palm (*Attalea maripa*). The most common species is *Elaeis guineensis*, which is commonly known as the African oil palm. The oil palm (*Elaeis guineensis*) trees of the experimental site were planted in 2014, and were in full growth in early 2020. The average economic lifespan of the oil palm is 25 to 30 years. Figure 3 shows the basic characteristics of an oil palm tree. To obtain more information about the tree specifications, 5 trees were chosen randomly from a line of trees, and their biological characteristics were measured (Table 1).

Since the trees were planted at the same time of the year, the deviation of the measured values was barely noticeable.

### 3.5. Measurement Equipment

LoRa is the short form of long-range, based on the principle of chirp spread spectrum (CSS) wireless modulation technology systems, which use the wider band of 125, 250, and 500 kHz. In CSS technology, a narrow-band signal spreads over a wider-band channel. The data rate and coverage change due to the change in SFs (SF7-SF12), where a higher SF causes a lower data rate and greater coverage, and vice versa [34]. Moreover, LoRa technology is robust against performance degradation due to harsh multipath [35] interface and Doppler effects, because of the frequency modulated LoRa chirps, which increase the receiver’s sensitivity. LoRa operates on license-free sub-gigahertz bands—for example, 433 MHz (Asia), 868 MHz (Europe), and 915 MHz (Australia). These frequencies fall into ISM bands, which are reserved internationally for industrial, scientific, and medical purposes. LoRa propagation loss is measured by the RSSI (received signal strength indicator) parameter. This parameter has been used in many works as a transmission quality indicator [36,37,38]. The measurement of received signal strength (RSS) is followed by various metrics (Table 2).

**Figure 4:**
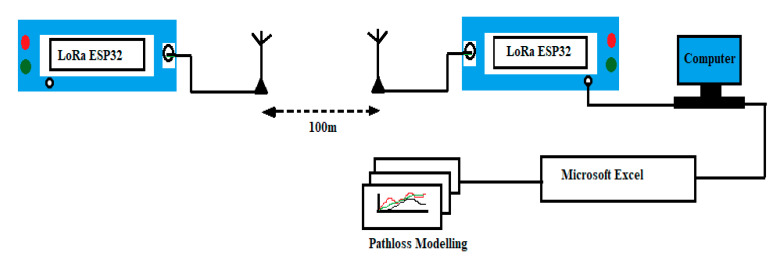
Measurement and modeling process diagram.

To conduct the measurement procedure, a pair of LoRa ESP32 devices (transmitter and receiver) was configured using Arduino software. Therefore, the modules were connected using power banks and placed at the desired height. The following process diagram (Figure 4) shows the entire measurement and modeling procedure.

The following table (Table 3) contains the detailed information about antenna heights for the RSS measurements.

### 3.6. Data Gathering Method

**Figure 5:**
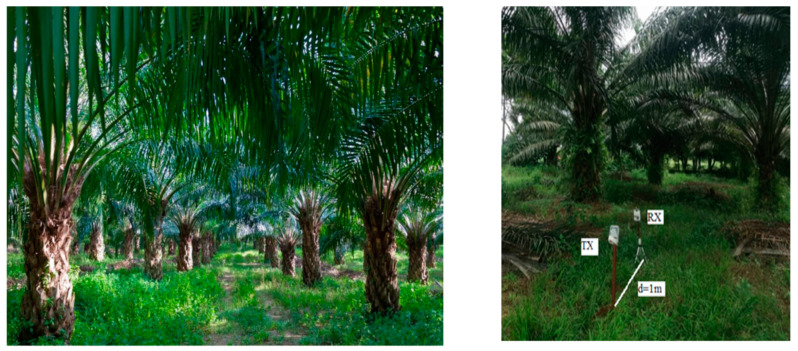
LOS links in between the two lines of trees (**left**); RSSI measurement (**right**).

The transmitter was programmed to send the data packet “hello” to the receiver every 100 microseconds. The measurement campaign was conducted in a few different scenarios in the plantation region to observe the effect of foliage on the propagation signal. The RSS data were collected for LoS links and NLoS links. For the LoS link measurements (Figure 5), the transmitter was fixed in one location, and the receiver was placed after 1 m, 2 m, 3 m, and so on, up to 100 m distance from the transmitter’s location. The exact same procedure was followed for each LoRa configuration.

The NLOS link measurement test was conducted in a line of oil palm trees using two approaches:

- Scenario 1: Propagation through the trunk.
- Scenario 2: Propagation through the canopy.

Moreover, another three sets of data were collected for validation of the prediction model for another line of trees, keeping the transmitter in the same place. The LoRa settings were maintained for different bandwidths and spreading factors. The LoRa settings used for path-loss prediction model validation were SF7_125 kHz, SF10_250 kHz, and SF12_500 kHz. The NLoS link was created in between two lines of trees, while the NLoS data were also collected for scenarios 1 and 2, as stated above.

## 4. Results and Discussion

### 4.1. Data Analysis and Prediction Model

This section attempts to analyze all of the data collected at the LoRa 433 MHz frequency, using three bandwidths and six spreading factors. Altogether, there are 18 LoRa parameter settings (6 settings for each bandwidth) for data collection, and about 1500 LoS (line-of-sight) and 300 NLoS (non-line-of-sight) propagation data are collected for path-loss prediction modeling.

To collect the data, the transmitter and the receiver were placed very near to the ground for LoS propagation, and for NLoS propagation the receiver’s height was 1.5 m, which is also very near to the ground. Therefore, the propagated signal underwent natural propagation effects such as scattering, reflection, diffraction, etc., resulting in degradation of the received signal’s strength with the increase in distance between the transmitter and the receiver. All of the empirically measured data analysis is presented for two segments: LoS link data, and NLoS link propagation data.

#### 4.1.1. LoS Link Data Analysis

The LoS link data were analyzed to find the path-loss exponent (*n*) for each LoRa setting using Equation (4). The path-loss exponent was calculated for all of the LoS measurements. The following graph represents the path-loss exponents (*n*) for the LOS measurements, and the average value of 100 path-loss exponents is taken as the value of the path-loss exponent of the corresponding settings. All of the path-loss exponent values are presented in Table 4. It is noticeable that the path-loss exponent decreases from SF7–SF12 with the change in the bandwidth. However, there is a notable exception in SF8 (BW 125 kHz and BW 250 kHz). The exception for the SF8 data behavior may be because of the environmental effects. Moreover, all the values are approximately similar for the BW 125 kHz and 250 kHz bandwidths. The values for the 500 kHz frequencies are always greater than those of the other two bandwidths. Therefore, from analyzing the data, the prediction value was found to be 2.34 for 125–500 kHz bandwidths and 2.9 for the 500 KHz bandwidth.

#### 4.1.2. NLoS Link Data Analysis

**Figure 6:**
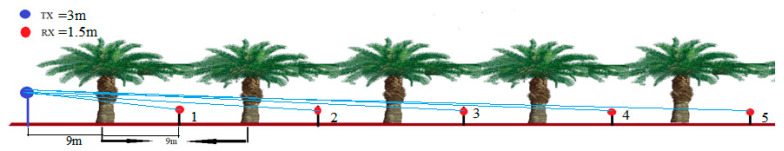

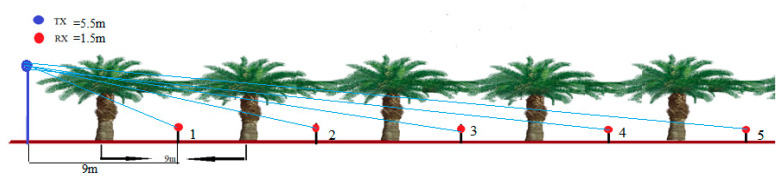
Scenario 1: propagation through the trunks (**above**); Scenario 2: propagation through the canopy (**below**).

The average intervening wall loss (per trunk and per canopy) was calculated for scenarios 1 and 2 (Figure 6) for all of the LoRa settings. The following table (Table 5) represents all the trunk and canopy attenuations.

The highest average intervening wall loss per trunk for LoRa propagation was found to be 7.66 dB, while the lowest was 4.98 dB. However, for RFID propagation in the palm oil plantation, the average trunk loss was 2.49 dB per tree [10]. This might be because of either the different propagation modulation types used by LoRa, or the differences in the physical characteristics of the oil palm trees. The trunk attenuation decreased in a similar pattern for all of the bandwidths, while for SF8 and SF9 there was a noticeable drop in the attenuation (2 dB and above). However, from SF9 to SF12, there were no significant average attenuation changes (<1 dB) observed.

Likewise, for the trunk attenuation, the data obtained from Scenario 2 also show a similar pattern to that of Scenario 1. The canopy attenuation depends on the spreading factor changes, and insignificant change was observed due to the change in the bandwidth. The highest attenuation was 9.56 dB and the lowest attenuation was 5.37 dB among all of the measurements

**Figure 7:**
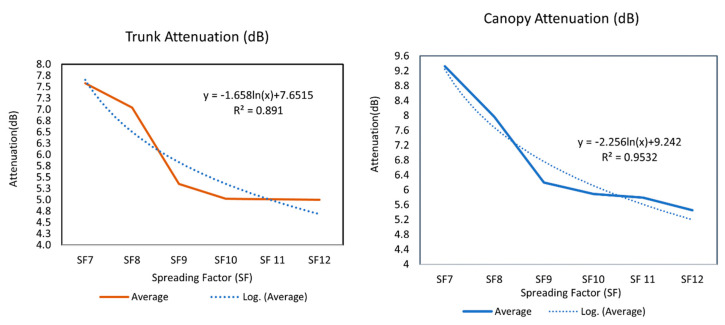
Trunk and canopy attenuation functions.

For every SF, the trunk and canopy attenuation changed greatly, and insignificant changes were observed with the change in bandwidth using the same spreading factor. Therefore, the trunk and canopy attenuation are not bandwidth-dependent functions; rather, they depend on the change in the spreading factor. Hence, the prediction model is a function of the spreading factor (see Figure 7). The graphs show the best fit of the data trend line based on the R-squared value. The R-squared is the coefficient of determination, which evaluates the scattered data points around the fitted curve. A value of R-squared that approaches 1 is considered a more accurate data trend line. To get the best fit for the trunk and canopy attenuation data, the highest R-squared value was considered among the popular curve-fitting trend lines (e.g., linear, exponential, logarithmic, and polynomial).

Choosing the highest R-squared value ($R^{2}=0.891)$ to investigate the best curve fitting, we found that the trunk attenuation changes in a logarithmic scale with the change in the spreading factor. The prediction function for trunk attenuation is as follows:

*y* = −1.658 *ln(x)* + 7.6515 (8)

Equation (8) denotes the change in the trunk attenuation (*y*) depending on the SF (*x*). The variables *x* = 1, 2, 3, 4, 5, and 6 are for the settings SF7, SF8, SF9, SF10, SF11, and SF12, respectively.

Again, all of the measured data from Scenario 2 were also analyzed, and the graph shows the canopy attenuation function by choosing the best fit of data with the highest $R^{2}=0.9532$ value (see Figure 6). The canopy attenuation also changes in a logarithmic scale with the change in the spreading factors. The prediction function for trunk attenuation is as follows:

(9) y=−2.256 ln(x)+9.242

Equation (9) denotes the change in the canopy attenuation (*y*) depending on the SF *(x*). The variables *x* = 1, 2, 3, 4, 5, and 6 are for the settings SF7, SF8, SF9, SF10, SF11, and SF12, respectively.

Therefore, combining all of the components of the proposed path-loss prediction model, the path-loss equation for 433 MHz in the palm oil plantation is as follows (Table 6):

(10) $$PL_{palm-oil\left(433\mathrm{MHz}\right)}=PL_{0}+10nlog\left(d\right)+\overset{N}{\underset{C=1}{\sum }}C_{N}+\overset{N}{\underset{T=1}{\sum }}T_{N}$$

#### 4.1.3. Validation of the Prediction Model

**Figure 8:**
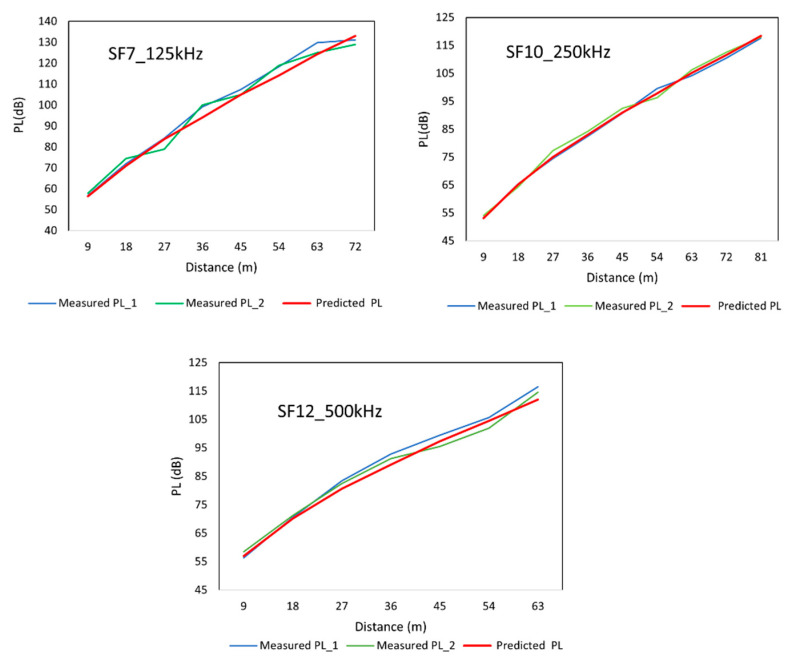
Comparison with Measured PL_2.

To validate the prediction model, 300 LoS and 30 NLoS data were collected to measure path loss (Measured PL_2). Three random LoRa settings were chosen for three bandwidths and spreading factors to compare with the reference measurement (Measured PL_1) and the path-loss prediction model (Figure 8).

From the above figure, the deviation of measured path loss from the prediction model can be observed to be insignificant (<3 dB). The change in the path-loss values for another line of trees can be the consequence of the multipath fading of the propagated signal when the signal is reflected, diffracted, and scattered in the propagation medium. However, the trees in a line are almost the same size and planted in a unique pattern; the biological structure of the oil palm plants with unevenly scattered leaves could also be the reason for this variation. Since there is no acute deviation observed, the prediction model is still sustainable for the palm oil plantation. Therefore, it can be concluded that the path-loss prediction model can predict the path loss for an approximately similar-sized and typical-patterned palm oil plantation.

### 4.2. PL Measurement Analysis

In this research, each measurement showed varying signal strength (RSS) data for each LoRa radio configuration. This is because of the natural propagation phenomena known as diffraction, refraction, and reflection of the transmitted signal, which are caused by the surroundings of the palm oil plantation environment [39].

Some excess attenuations can be caused due to losses from atmospheric factors such as breezing wind. Since the measurements were carried out in the dry season, the effects of rainfall were not considered in this research. However, researchers have found that rainfall and atmospheric or gaseous absorption at frequencies below 10 GHz have insignificant effects on signal propagation [40]. Therefore, this research concerning the 433 MHz frequency might not be subject to rainfall and other atmospheric losses, such as humidity and temperature.

Taking the measurements for the line-of-sight propagation path, the electromagnetic wave passes through several obstacles—including grass, weeds, and uneven ground—in the palm oil plantation, resulting in signal loss; a varied path-loss exponent is given in Table 4. Moreover, the multipath fading that occurred in the palm oil plantation was not ass static as it may seem. This fluctuation may be caused by the dynamic movement of grass and weed petals present on the plantation surface [41].

Moreover, variation in the range was noticed for different spreading factors and bandwidth settings when conducting the experiments in non-line-of-sight scenarios (line of oil palm trees). It was found that every measurement point obstacle presented by the oil palm trees showed distinct measurement results (RSS values) compared to the other nearby line-of-sight measurement points. Switching the bandwidth from 125 to 500 kHz changed the range; however, the 250 kHz bandwidth showed similar ranges to the 125 kHz bandwidth.

Again, in 100 m distance, SF7–SF12 were able to penetrate 7–10 oil palm trees while using 125–250 kHz bandwidth. However, the 500 kHz bandwidth setting was capable of penetrating 5–7 oil palm trees. Therefore, the non-line-of-sight measurements of the palm oil plantation can be concluded as follows:

- Obstacle loss due to the oil palm trees in the plantation region degraded the LoRa signal quality. As demonstrated by Petäjäjärvi et al., increasing the bandwidth decreases the communication range and sensitivity [42]. This was also witnessed in the palm oil plantation propagation medium.
- Obstacle loss in the palm oil plantation was caused by two main parts of the oil palm tree (i.e., trunk and canopy). Due to the symmetric pattern of the palm oil plantation and similarity in the tree structure, the path loss did not fluctuate drastically. Therefore, the path-loss prediction is generally easier in palm oil plantation scenario.

### 4.3. Comparison with Empirical Path-Loss Models

**Figure 9:**
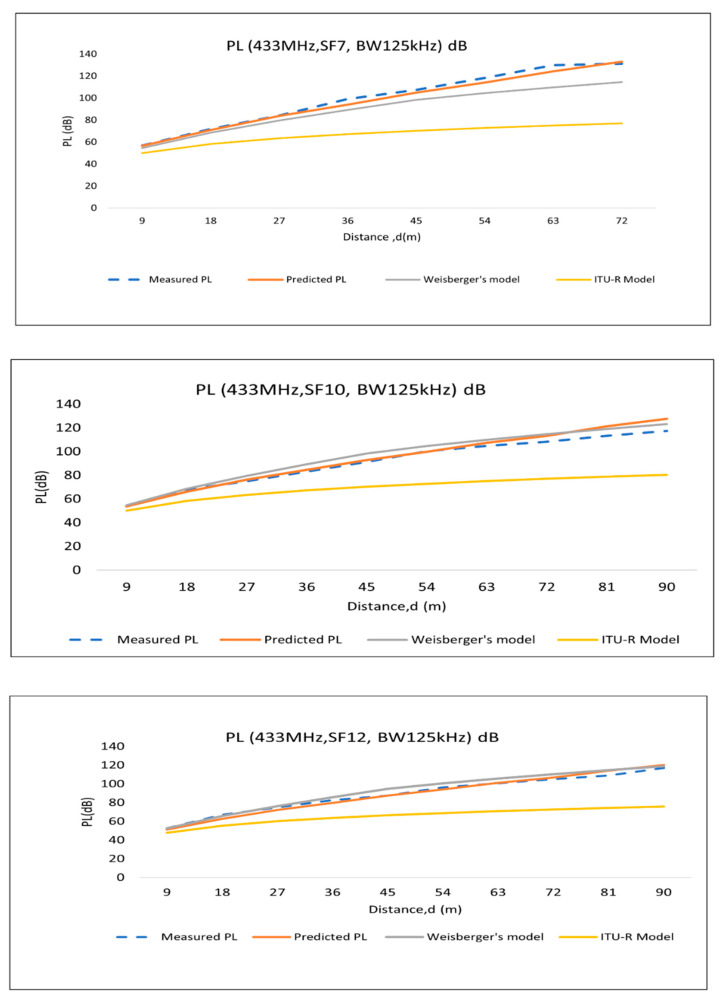
Path-loss comparison of 125 kHz bandwidth LoRa settings.

**Figure 10:**
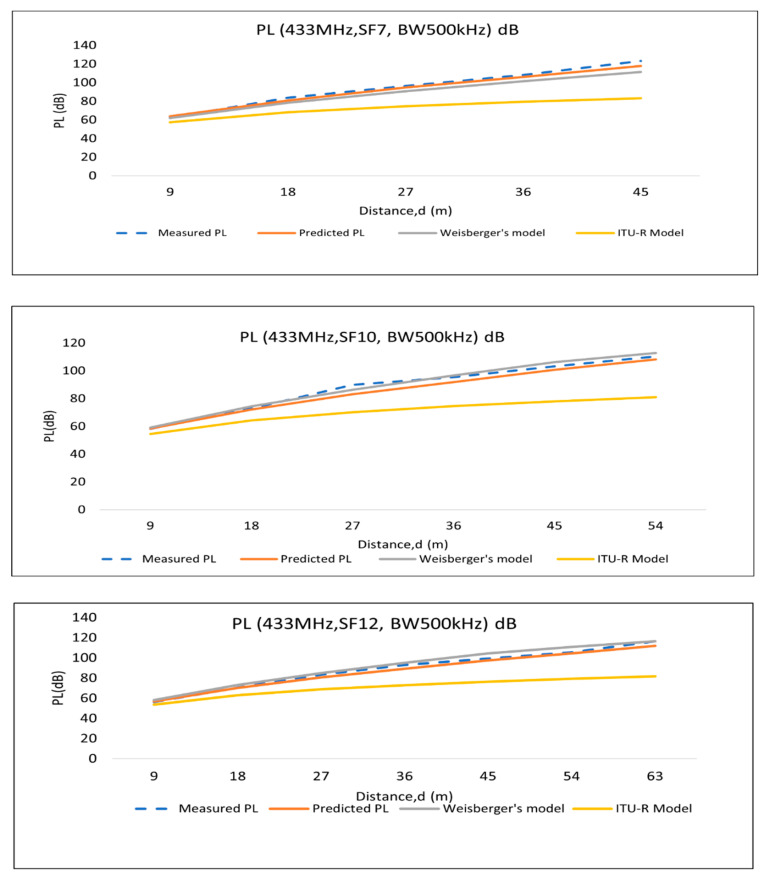
Path-loss comparison of 500 kHz bandwidth LoRa settings.

Considering the dense canopy loss in the empirical foliage path-loss models in this research, the proposed path-loss model for Scenario 2 (Figure 5) includes trunks and canopies in the propagation path. Therefore, in the existing foliage loss models, we consider 30% foliage depth in between TX and RX. Hence, the foliage depth (d = 0.3 d) is considered for the abovementioned foliage loss models. The following graphs (Figure 9 and Figure 10) represent the path-loss comparisons, which are discussed below.

In this study, RSS data were collected for 433 MHz LoRa channels and analyzed. Since there are similarities between the measured data of BW 125 kHz and BW 250 kHz, among these two BWs, 125 kHz data were chosen for the comparison. Therefore, the comparison is presented for BW 125 kHz and BW 500 kHz. Again, SF7, SF10, and SF12 data are used for comparison to see the path-loss models’ behavior.

As shown in the graphs (Figure 9 and Figure 10), the predicted model deviates from the measured path loss (proposed model) because the prediction model is constructed using the averaged values for trunk and canopy attenuation. However, in real environment, all of the canopies and trunks are not the same in size, and the attenuation does not remain constant over the distance in a line of oil palm trees. Again, it is noticeable that Weissberger’s model is very near to the model proposed in this research, whereas the ITU-R model deviates largely from the proposed and predicted PL models.

To observe the deviation of the multiwall prediction model from the measured and empirical models, RMSE (root-mean-square error) was calculated, since other researchers have also adopted RMSE as a validation method for path-loss propagation evaluation [39]. The RMSE (root-mean-square error) values present the variation between the empirical measurements and predicted/related empirical models for the palm oil plantation environment. RMSE is the mean value of the mean of the square of all of the error; RMSE for path loss (dB) is calculated as follows (Table 7):

(11) $$\mathrm{RMSE}=\sqrt{\frac{\sum_{i=1}^{N}{\left(\mathrm{Measured}\;\mathrm{PL}-\left(\mathrm{Predicted}\;\mathrm{or}\;\mathrm{Weissberger}\;\mathrm{or}\;\mathrm{ITU}\_R\right)\;\mathrm{PL}\right)}^{2}}{N}}$$

From the RMSE values, it is noticeable that the prediction model is the closest (mean RMSE 2.74 dB) to the measured PL; however, for SF10, BW 500 kHz LoRa channel propagation PL is close (mean RMSE 5.32 dB) to Weissberger’s model. The ITU-R foliage loss model shows the maximum RMSE (mean 23.24 dB) with respect to the measured PL. Therefore, the ITU-R foliage loss model cannot predict the path loss for palm oil plantations.

## 5. Conclusions and Future Work

In this research, LoRa propagation was analyzed using the empirical measurements taken at a 433 MHz frequency in a palm oil plantation in Selangor, Malaysia. The LoS measurements were used to find the path-loss exponent (*n*), where the spreading factors (SFs) show the different propagation characteristics; hence, there was a noticeable change in the value of the path-loss exponent (n). The averaged prediction value of the path-loss exponent for BW 125–250 kHz and 500 kHz bandwidths were found to be 2.37 and 2.9, respectively. Again, the NLoS measurements were used to find the trunk and canopy loss prediction functions. Moreover, in the comparison of the predicted path loss and the existing empirical path-loss models (Weissberger’s and ITU-R), the prediction model was the more accurate (average RMSE 2.74) with respect to the measured path loss. However, Weissberger’s model was close in prediction of the path loss in the palm oil plantation (average RMSE 5.32), whereas the ITU-R foliage model was far from predicting the path loss in the palm oil plantation.

The prediction model shows good performance with respect to the reference measured data, and this model is a site-specific model for palm oil plantations. However, the model could be applicable for other foliage propagation as well, provided that the plantation maintains the unique pattern typical of modern palm-oil plantations. As with any other wave propagation, LoRa signal propagation also undergoes multipath fading due to scattering, reflection, and diffraction. Furthermore, due to the unevenly scattered leaves, the path-loss results show a slight variation from other measurements taken in other lines of trees. This research is based on empirical measurements, and the path-loss modeling is not sustainable for heavy rainfall and harsh weather conditions. However, for the further improvement of the prediction model, numerous measurements from different-sized oil palm trees could be undertaken and analyzed with software-based modeling platforms.

The prediction model incorporated a 433 MHz LoRa frequency; other frequency channels could also be utilized to form multiwall and multi-frequency path-loss prediction models. To achieve higher accuracy of the prediction model, more research should be conducted in other palm oil plantations in Malaysia and other tropical locations to improve the path-loss prediction model by using the correction factors for the distinct trunk and canopy sizes of oil palm trees.

## Figures and Tables

**Table 1:** Measurement of oil palm tree characteristics.

Oil Palm Tree	Tree Height (m)	Trunk Height (m)	Trunk Diameter (m)	Canopy Depth (m)	Canopy Diameter (m)
Tree 1	6.50	3.50	0.64	3.00	9.00
Tree 2	6.72	3.52	0.64	3.22	9.31
Tree 3	6.65	3.65	0.71	3.01	9.32
Tree 4	6.72	3.71	0.79	3.51	9.57
Tree 5	7.35	3.85	0.79	3.52	9.51
Average	6.79	3.65	0.714	3.25	9.34

**Table 2:** LoRa parameter settings.

Parameter	Value
Frequency	433 MHz
Bandwidth (BW)	125 kHz, 250 kHz, 500 kHz
Spreading Factor (SF)	SF7, SF8, SF9, SF10, SF11, SF12
Antenna Gain	2 dBi
Tx-Power	14 dbm
Coding Rate (CR)	4/5
Output power	14 dBm

**Table 3:** Antenna height setup.

Measurement Type	Area	Height (Tx)m	Height (Rx)m	Initial Distance between Tx-Rx (m)
Line-of-Sight (LoS)	Open Space	1	1	1
Non-Line-of-Sight (NLoS)	Trunk	3	1.5	9
Non-Line-of-Sight (NLoS)	Canopy	5.5	1.5	9

**Table 4:** Path-loss exponent (*n*) prediction value.

Path-Loss Exponent, *n*					
SFs	BW 125 kHz	BW 250 kHz	Average	BW 500 kHz	Average
SF7	2.37	2.36	2.34	3.15	2.9
SF8	2.63	2.52		2.98	
SF9	2.39	2.44		2.87	
SF10	2.37	2.33		2.84	
SF 11	2.12	2.26		2.71	
SF12	2.14	2.16		2.74	

**Table 5:** Trunk and canopy attenuation.

	Trunk Attenuation (dB)				Canopy Attenuation (dB)			
SFs	125 kHz	250 kHz	500 kHz	Avg.	125 kHz	250 kHz	500 kHz	Avg.
SF7	7.5	7.57	7.66	7.58	8.98	9.56	9.42	9.32
SF8	7	7	7.11	7.04	7.85	7.99	8.04	7.96
SF9	5.5	5.5	5.04	5.35	5.94	6.24	6.43	6.2
SF10	5	5	5.06	5.02	5.62	5.72	6.33	5.89
SF11	5	5.05	4.98	5.01	5.7	5.65	6.01	5.79
SF12	5	5	5.01	5.00	5.5	5.47	5.37	5.45

**Table 6:** PL prediction model for 433 MHz.

Components	Function
$PL_{0}$	20logf−27.55, *f* = 433 MHz
*n*	2.34 (BW 125 to 250 kHz)
	2.9 (BW 500 kHz)
T	*y* = −1.658 *ln(x)* + 7.6515
C	*y* = −2.256 *ln(x)* + 9.242

**Table 7:** RMSE comparison values.

LoRa Channels	RMSE (dB)		
433 MHz	Predicted	Weissberger	ITU-R
SF7, BW 125 kHz	3.24	10.73	34.1
SF10, BW 125 kHz	2.04	4.75	19.12
SF12, BW 125 kHz	2.07	3.9	20.08
SF7, BW 500 kHz	3.10	7.04	25.02
SF10, BW 500 kHz	3.39	2.32	20.02
SF12, BW 500 kHz	2.62	3.19	21.1
Avg.	2.74	5.32	23.24

## Data Availability

Not applicable.
